# Human amniotic epithelial cells ameliorate kidney damage in ischemia-reperfusion mouse model of acute kidney injury

**DOI:** 10.1186/s13287-020-01917-y

**Published:** 2020-09-23

**Authors:** Yifei Ren, Ying Chen, Xizi Zheng, Hui Wang, Xin Kang, Jiawei Tang, Lei Qu, Xiaoyan Shao, Suxia Wang, Shuangling Li, Gang Liu, Li Yang

**Affiliations:** 1 Renal Division, Peking University First Hospital, Peking University Institute of Nephrology, Beijing, 100034 People’s Republic of China; 2 Key Laboratory of Renal Disease, Ministry of Health of China, Beijing, 100034 People’s Republic of China; 3 Key Laboratory of Chronic Kidney Disease Prevention and Treatment (Peking University), Ministry of Education, Beijing, 100034 People’s Republic of China; 4 Renal Pathology Center, Peking University First Hospital, Beijing, 100034 People’s Republic of China; 5 Laboratory of Electron Microscopy, Pathological Center, Peking University First Hospital, Beijing, 100034 People’s Republic of China; 6 Shanghai iCELL Biotechnology Co Ltd., Shanghai, 200333 People’s Republic of China; 7 Department of Critical Care Medicine, Peking University First Hospital, Beijing, 100034 People’s Republic of China

**Keywords:** Acute kidney injury, Human amnion epithelial cells, Exosome, Cell therapy, Ischemia

## Abstract

**Background:**

Acute kidney injury (AKI) is a common clinical disease with complex pathophysiology and limited therapeutic choices. This prompts the need for novel therapy targeting multiple aspects of this disease. Human amnion epithelial cell (hAEC) is an ideal stem cell source. Increasing evidence suggests that exosomes may act as critical cell–cell communicators. Accordingly, we assessed the therapeutic potential of hAECs and their derived exosomes (hAECs-EXO) in ischemia reperfusion mouse model of AKI and explored the underlying mechanisms.

**Methods:**

The hAECs were primary cultured, and hAECs-EXO were isolated and characterized. An ischemic-reperfusion injury-induced AKI (IRI-AKI) mouse model was established to mimic clinical ischemic kidney injury with different disease severity. Mouse blood creatinine level was used to assess renal function, and kidney specimens were processed to detect cell proliferation, apoptosis, and capillary density. Macrophage infiltration was analyzed by flow cytometry. hAEC-derived exosomes (hAECs-EXO) were used to treat hypoxia-reoxygenation (H/R) injured HK-2 cells and mouse bone marrow-derived macrophages to evaluate their protective effect in vitro. Furthermore, hAECs-EXO were subjected to liquid chromatography-tandem mass spectrometry for proteomic profiling.

**Results:**

We found that systematically administered hAECs could improve mortality and renal function in IRI-AKI mice, decrease the number of apoptotic cells, prevent peritubular capillary loss, and modulate kidney local immune response. However, hAECs showed very low kidney tissue integration. Exosomes isolated from hAECs recapitulated the renal protective effects of their source cells. In vitro, hAECs-EXO protected HK-2 cells from H/R injury-induced apoptosis and promoted bone marrow-derived macrophage polarization toward M2 phenotype. Proteomic analysis on hAECs-EXO revealed proteins involved in extracellular matrix organization, growth factor signaling pathways, cytokine production, and immunomodulation. These findings demonstrated that paracrine of exosomes might be the key mechanism of hAECs in alleviating renal ischemia reperfusion injury.

**Conclusions:**

We reported hAECs could improve survival and ameliorate renal injury in mice with IRI-AKI. The anti-apoptotic, pro-angiogenetic, and immunomodulatory capabilities of hAECs are at least partially, through paracrine pathways. hAECs-EXO might be a promising clinical therapeutic tool, overcoming the weaknesses and risks associated with the use of native stem cells, for patients with AKI.

## Introduction

Acute kidney injury (AKI) is a common clinical disorder, which is defined as an abrupt decline in kidney function [1]. Renal ischemia reperfusion (I/R) is one of the major causes of acute kidney injury (IRI-AKI), with high morbidity and mortality [2]. Clinically, ischemia may result from a variety of conditions, such as the use of vasoconstrictive drugs or radiocontrast agents, decreased cardiac output after surgery and trauma, renal vascular occlusion or obstruction, and kidney transplantation [3]. Ischemia and hypoxia cause tubular and endothelial necrosis and apoptosis, accompanied by cytokines, chemokines, and reactive oxygen species (ROS) release. These signals initiate the infiltration of professional inflammatory cells such as neutrophils and monocytes, leading to enhanced inflammation and further cell injury and destruction [4]. Additionally, endothelium injury and microvascular plugging mediated by adherent leukocytes result in peritubular capillary loss which worsens ischemia [5]. Due to the multi-factorial pathophysiological mechanisms, once established, there is no effective pharmacological method to prevent IRI-AKI development or to reverse tissue injury [6]. Therefore, novel and effective therapies are urgently needed.

In the past few years, stem cell therapy has been recognized as a potential treatment strategy for AKI [7]. Human amniotic epithelial cell (hAEC), derived from the epithelial layer of amniotic membrane, is a stem cell that possesses embryonic stem cell-like differentiation capability and adult stem cell-like immunomodulatory properties [8]. hAECs have attracted widespread attention in regenerative medicine due to its pluripotency, low mutation frequency, low immunogenicity, low tumorigenicity, rich sources, and no ethical risks [8]. Because of these advantageous characteristics, hAECs have obtained a good outcome after transplantation in many diseases, including lung injury [9], brain injury [10], and hepatic fibrosis [11]. However, it is unclear whether hAECs have any therapeutic potential on acute kidney injury.

It has been observed that stem cell therapy has beneficial renal protection in a variety of kidney pathologies, yet stem cells have not been detected to differentiate into a sufficient number of cells to reconstruct renal parenchymal organs [12, 13]. Therefore, studies have increasingly focused on the paracrine action of stem cells. Almost all cell types including stem cells secret extracellular vesicles (EVs) either from plasma membrane shedding (microvesicles) or from intracellular multivesicular bodies releasing (exosomes) [14]. Exosomes are smaller in size (30–150 nm) compared to microvesicles (0.1 to 1 μm) and differ in antigenic composition [15]. Exosomes are important information carriers regulating both cellular function and gene expression via transferring cargos such as lipids, proteins, mRNA, microRNAs, and non-coding RNAs into recipient cells [16]. Recent studies have reported that hAEC-derived exosomes (hAECs-EXO) can reduce liver fibrosis [17], restrict lung injury and enhance endogenous lung repair in bleomycin-challenged aged mice [18]. Clinically, the use of exosomes may have some advantages over their source cell, such as being convenient to extract, store, and transport; easy to be internalized into targeted cells; lower in immunogenicity; and better in biocompatibility [19]. It has been reported that preconditioning of the isolated kidney with MSC-derived extracellular vesicles before transplantation could limit tissue damage due to ischemia-reperfusion injury and chronic allograft nephropathy [20].

In this study, we aimed to explore whether hAECs or their derived exosomes can reduce mortality and improve renal function in an IRI-AKI mouse model. The potential therapeutic mechanisms were further investigated.

## Material and methods

### Mice

Male C57BL/6j mice (9–10 weeks) were purchased from Vital River Laboratory Animal Technology (Beijing, China) [License No. SCXK (Jing) 2016-0011]. All mice were maintained in animal facilities under SPF conditions. All animal experiments were performed with the approval of the Institutional Animal Care and Use Committee of Peking University First Hospital (Approval Number: J201868).

### Culture of hAECs

The human amniotic epithelial cells were provided by Shanghai iCELL Biotechnology Co., Ltd. (Shanghai, China). Briefly, the human amniotic epithelial cells were isolated from fresh amnion membranes collected from healthy mothers after cesarean deliveries with written and informed consent. The procedure was approved by the Institutional Ethics Committee of the International Peace Maternity and Child Health Hospital, School of Medicine, Shanghai Jiao Tong University (Approval Number: [2014]11). The amnion was peeled from the collected placentas; then, the blood and mucus were washed away with PBS carefully. The collected amnion tissue was then transferred into a 150-ml flask and dissociated with 50 ml 0.25% trypsin-EDTA for 30 min at 37 °C. The digestive process was ended by adding 100-ml culture medium (DMEM/F12 (Gibco, Grand Island, NY, USA) with 10% fetal bovine serum (FBS, Gibco, USA)). The cell suspension was transferred through a 200-mesh stainless steel screen, centrifuged at 650*g* for 5 min. The cell pellet was then suspended in fresh culture medium. The cultured P1-hAECs were then frozen in CELLBANKER (ZENOAQ, Fukushima, Japan) at a concentration of 5 × 10^6^/ml and stored in a refrigerator at − 80 °C overnight. Then, frozen vials were transferred into liquid nitrogen for long-term preservation. Before transplantation, hAECs were thawed by gently agitating the vial in a 37 °C water bath. Cells were seeded onto 100-mm plates containing complete culture medium in 5% CO_2_ incubator at 37 °C. For hAEC transplantation, the confluent hAECs were digested with 0.25% trypsin-EDTA at 37 °C and then resuspended in phosphate-buffered solution (PBS) at 10^7^/ml. hAECs used in the experiments had undergone fewer than two passages.

### Exosome isolation and characterization

Exosomes were obtained from the supernatants of hAECs through ultracentrifugation according to classical methods reported previously [21]. In brief, the complete culture medium from hAECs was collected and centrifuged at 2000*g* for 30 min and filtered through a 0.22-μm cell filter (Millipore, Billerica, MA, USA) to remove debris. The cell-free medium was then centrifuged at 20,000*g* (Beckman Coulter, USA) for 30 min at 4 °C to remove micro-vesicles. The supernatants were discarded, and the pellets were washed in PBS, after which the suspension was centrifuged at 100,000*g* for 70 min at 4 °C to obtain exosomes. The ultrastructure of the exosomes was analyzed under a transmission electron microscope (Zeiss, Oberkochen, Germany). The protein levels of exosome markers CD63, TSG101, Alix, and Flotllin were detected using western blot. To determine the size and concentration of the purified vesicles, a nanoparticle tracking analysis (NTA) was performed using Zetaview software (Particle Metrix, Meerbusch, Germany).

### Induction of IRI-AKI

Ischemia for 30–33 min at 37 °C was induced in both kidneys using the flank approach as previously reported [22]. Briefly, 9- to 10-week-old C57BL/6j male mice (25–27 g) were anesthetized with an intraperitoneal injection of pentobarbital sodium (5 mg/ml pentobarbital in saline, 50–60 mg/kg). The mouse was placed on the thermostatic station lying on prone position. The body temperature was monitored through a rectal probe and controlled in the range of 36.9–37.1 °C throughout the surgery. The incision on each side was positioned at 1/3 of the body from the back of the mouse, and the incision size was 1–1.5 cm along the back. The kidneys were exposed, and both renal pedicles were clamped for 30 or 33 min using nontraumatic microaneurysm clips (Roboz Surgical Instrument Co.). Complete ischemia was indicated by color change of the kidney from red to dark purple in a few seconds. The ischemic time of each side was recorded separately to ensure both kidneys receiving the same durations of ischemia. After ischemia, the micro-aneurysm clips were released for each kidney to start the reperfusion, which was indicated by the change of kidney color to red. All the mice received 1 ml of warm saline intraperitoneally at the end of the surgery. The animal was kept on a heating pad until it gained full consciousness before being transferred to its housing cage. Sham-operated mice underwent the same procedure, except for the clamping of renal pedicles.

One hundred microliters of cell-free vehicle (PBS), hAECs (1 × 10^6^ cells), or hAECs-EXO (about 3 × 10^8^ exosomes) in vehicle was injected into injured mice intravenously at the end of the procedure. The dosage of hAECs (1 × 10^6^ cells/mouse) in the treatment group was determined by the toxicity test and based on previous studies that used mesenchymal stem cells in murine IRI models [23, 24]. For toxicity test of hAECs on C57BL/6 by intravenous injection, three dose escalations, 2.5 × 10^7^ cells/kg, 5.0 × 10^7^ cells/kg, and 1.0 × 10^8^ cells/kg, were examined, respectively. The maximum tolerated dose was 1.25 × 10^6^ cells/25 g mouse. Through NTA analysis, usually 1 × 10^6^ hAECs could produce about 3 × 10^8^ exosomes.

On day 1, day 2, day 3, and day 7 post-surgery, the animals were euthanized with an overdose of pentobarbital sodium. The heart was perfused with 10 ml of ice-cold PBS before the organs were collected for subsequent experiments. All animal procedures were approved by the Institutional Animal Care and Use Committee of Beijing and were performed in accordance with the National Research Council Guide for the Care and Use of Laboratory Animals. Efforts were made to minimize animal suffering and limit the number of animals used in the study.

Plasma creatinine was determined by the picric acid method as previously reported [25]. Kidney tissue sections were fixed with 10% buffered formalin followed by paraffin embedding and stained with periodic acid-Schiff (PAS). The degree of tubulointerstitial damage was scored semi-quantitatively by a renal pathologist who was blinded to the experimental groups. The scores were based on a 0 to 4+ scale, according to the percentage of the cortex and medullar junction region affected by loss of brush border and tubular necrosis and/or apoptosis (0 = no lesion, 1+ = < 25%, 2+ = > 25 to 50%, 3+ = > 50 to 75%, 4+ = > 75 to < 100%).

### RNA extraction, reverse transcription, and real-time RT-PCR

A fraction of kidney was harvested, and RNA was isolated using TRIzol® reagent following the manufacturer’s instructions (Ambion, Thermo Fisher Scientific Inc., Waltham, MA, USA). RNA concentrations were determined by photometric measurements. cDNA was synthesized from 2 μg total RNA using FastKing RT Enzyme (KR118; TIANGEN Biotech, Beijing, China) for real-time RT-PCR. The primers used for PCR analyses were listed in Supplementary Table 1. Real-time PCR reagents were prepared from the SYBR Green PCR Master Mix (FP209; TIANGEN Biotech, Beijing, China), and all PCR analyses were performed on an ABI Vii7 system.

### Immunohistochemistry staining

Immunohistochemistry staining of the kidney was performed on paraffin sections. The primary antibodies included rabbit anti-Ki67 (1:400, Cat. No. 9129, Cell Signaling Technology) and rabbit anti-CD31 (1:100, Cat. No.28364, Abcam). The slides were then exposed to DAB-labeled secondary antibodies. The staining was examined using microscope (Leica, Germany). The number of positive cells was counted in 7–8 high-powered fields from the outer medulla in each kidney examined. Apoptosis in kidney tissues was detected on paraffin sections by the in situ terminal deoxynucleotidyl transferase-mediated dUTP nick end-labeling (TUNEL) method following the standard protocol (Beyotime, China).

### HK-2 cell hypoxia-reoxygenation (H/R)

HK-2 (immortalized human proximal tubular cells) cells were cultured in DMEM (Gibco) with 10% FBS (Gibco) and 1% penicillin and streptomycin (Gibco) under a humidified atmosphere consisting of 5% CO_2_ and 95% air at 37 °C (control group). Hypoxia-reoxygenation (H/R) injury was introduced by exposing the cells to hypoxic conditions (1% O_2_, 5% CO_2_, and 94% N_2_) for 48 h, followed by reoxygenation under normoxic conditions (5% CO_2_ and 95% air, reoxygenation) for 24 h in DMEM medium with 10% FBS (H/R group). A concentration of 1 × 10^8^/ml hAECs-EXO was added to DMEM culture medium before H/R injury for the H/R+EXO group.

### Western blot analysis

Samples were lysed on ice in lysis buffer supplemented with protease inhibitors. Aliquots of cell lysates were boiled in SDS-PAGE sample buffer, fractionated on 12% SDS-PAGE gel, and transferred to PVDF membrane. The membranes were blocked with 5% milk in TBST (Tris-buffered saline, 10 mM Tris-HCl [pH 7.5], 150 mM NaCl, and 0.1% Tween-20) for 1 h at room temperature and incubated with primary antibodies at 4 °C overnight. The following primary antibodies and dilutions were used: rabbit anti-CD63 (1:1000, Cat. No.134045, Abcam), rabbit anti-Alix (1:5000, Cat. No.186429, Abcam), rabbit anti-TSG101 (1:1000, Cat. No.125011, Abcam), rabbit anti-Flotillin (1:5000, Cat. No.133497, Abcam), rabbit anti-CD31(1:500, Cat. No.28364; Abcam), mouse anti-PCNA (1:1000, Cat. No.29, Abcam), rabbit anti-cleaved caspase3(1:1000, Cat. No.9661, Cell Signaling Technology), rabbit anti-Caspase3(1:1000, Cat. No.9662, Cell Signaling Technology), and anti-GAPDH (1:10000; Beyotime). Then, the membranes were incubated with horseradish peroxidase-conjugated secondary antibodies (1:1000; Beyotime) for 1 h at room temperature. Visualization of the blots was performed using the standard protocol for electrochemiluminescence (ECL; Santa Cruz Biotechnology). The relative intensity of the protein bands was quantified by digital densitometry using ImageJ software (NIH, Bethesda, MD, USA). The level of glyceraldehyde 3-phosphate dehydrogenase (GAPDH) was used as an internal standard.

### Cytokine and chemokine quantification

Bio-Plex Pro Mouse Cytokine 23-plex Assay (Bio-Plex Pro, Bio-Rad Laboratories, Inc.) was used to quantify the concentrations of 23 cytokines and chemokines in mouse kidneys: IL-1α, IL-1β, IL2, IL3, IL4, IL5, IL6, IL9, IL10, IL12/p40, IL12/p70, IL13, IL17A/CTLA8, Eotaxin/CCL11, G-CSF, GM-CSF, IFNγ, KC/CXCL1, MCP-1 (MCAF)/CCL2, MIP-1α/CCL3, MIP-1β/CCL4, RANTES/CCL5, and TNFα. Measurements were made on MAGPIX multiplexing instrument (Luminex Corporation, Austin, TX, USA). The manufacturer’s recommended quality control procedures were followed to ensure validity.

### Flow cytometry

Mice were anesthetized, sacrificed, and perfused with ice-cold PBS via the left ventricle for 2 min. Kidneys were minced and incubated with collagenase type IA (1 mg/ml, Sigma-Aldrich) in DMEM/F12 for 25 min at 37 °C with constant shaking. Digestion was stopped by the addition of ice-cold FBS. The digested kidney tissue suspension was passed through a 100-μm cell strainer (Thermo Fisher) and centrifuged at 1000*g* for 5 min at 4 °C. The pellet was then incubated with ACK Lysing Buffer (0.15 M NH4Cl, 10 mM KHCO3, and 0.1 mM Na2 EDTA) for 5 min at room temperature to remove red blood cells and centrifuged twice at 1000*g* for 5 min at 4 °C. The pellet was then washed with PBS. Cells were incubated with F4/80 (PE/cy7, Cat. No.123113, BioLegend) and CD206 (AF647, Cat. No. 141712, BioLegend) antibodies. Data were acquired using BD FACSVerse and analyzed using FlowJo software.

### Culture of mouse bone marrow-derived macrophage

L929 fibrosarcoma cells were cultured in RPMI 1640 (Gibco, UK) medium supplemented with 10% fetal bovine serum (Gibco, USA) at 37 °C in humidified air containing 5% CO_2_ for 48 h. L929 supernatant (1 × 10^6^ cells/mL) was collected and filtrated through 0.22-μm Millipore membrane and used in macrophage cultures (L929 sup).

Bone marrow-derived macrophages were isolated and cultured as described previously [26]. In brief, bone marrow cells were collected from the tibia and femur of 8-week-old C57BL/6 J mice. Cells were cultured in L929 conditioned medium (RPMI-1640 [Gibco, UK] with 10% heat inactivated fetal bovine serum [Gibco, USA], 15% L929 sup, and 100 U/mL penicillin-streptomycin [Gibco, UK]) and allowed to attach for 2 days. A fraction of cells was collected before polarization. The rest of cells were continued to culture in hAECs-EXO conditioned medium (RPMI-1640[Gibco, UK] with 10% heat inactivated fetal bovine serum [Gibco, USA], 1 × 10^8^ exosomes/ml, and 100 U/mL penicillin-streptomycin [Gibco, UK]) for 7 days. On day 8, the media was removed and the cells were lysed in TRIzol® reagent and quantified M1 and M2 macrophage marker gene expression by reverse transcription-PCR. The primers used for PCR analyses are listed in Supplementary Table 1.

### Statistical analysis

Statistical analysis was performed using SPSS 25.0 statistical software (SPSS, Chicago, IL). Normally distributed variables are expressed as mean ± standard deviation and compared using a *t* test. Non-normally distributed non-parametric variables are expressed as median and interquartile range and are compared between groups using the Mann-Whitney *U* test. All *P* values were two-tailed, and *P* < 0.05 was considered statistically significant.

## Results

### hAECs alleviated IRI-AKI

**Fig. 1:**
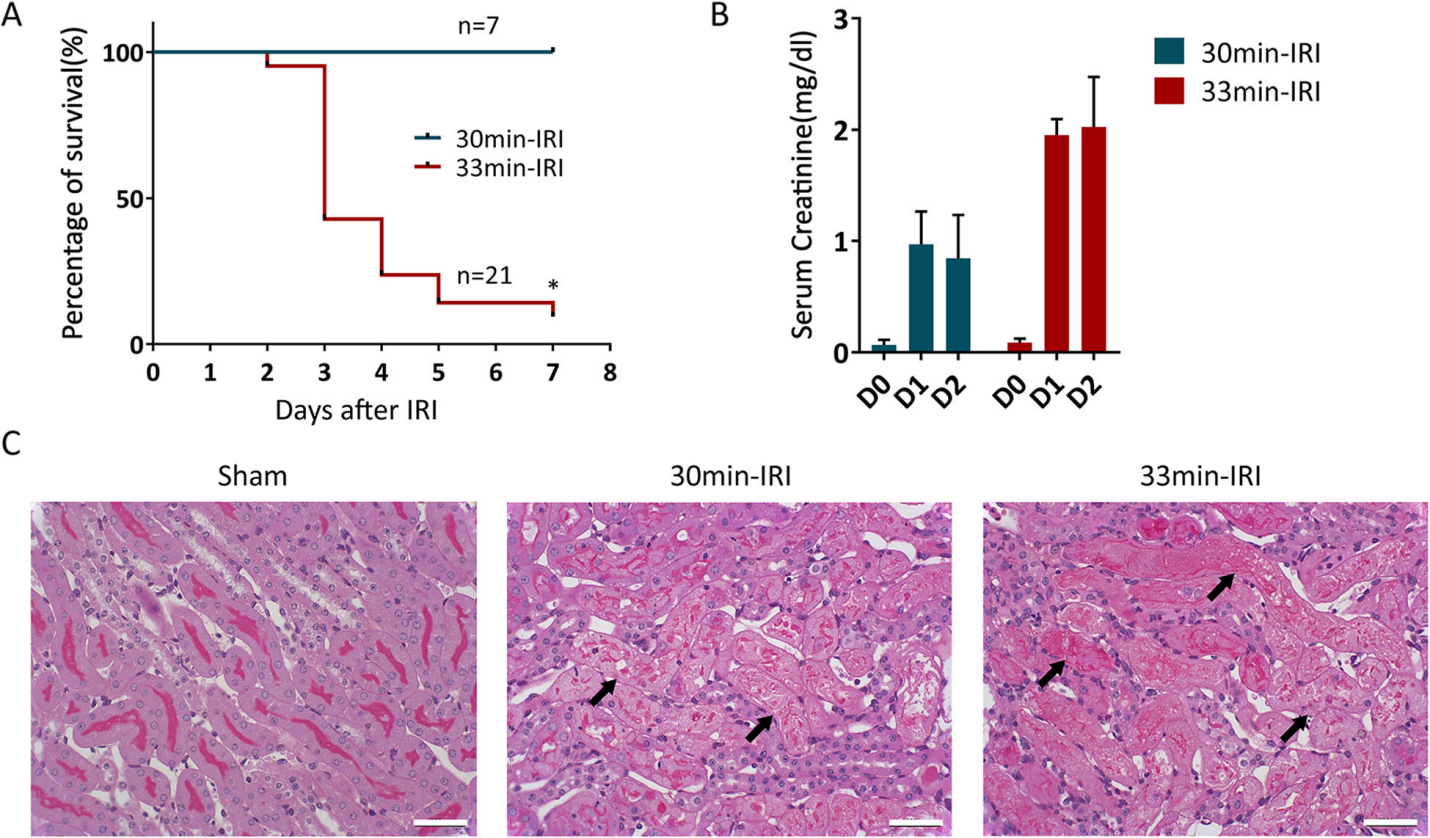
Establishment of IRI-AKI mouse model. **a** 7-day mortality rate in IRI mice with 30 min (30min-IRI, *n* = 7) or 33 min (33min-IRI, *n* = 21) ischemia. *P < 0.05. **b** Serum creatinine concentrations at day 1 and day 2 post-surgery in 30min-IRI group and 33min-IRI group. **c** Representative micrographs of PAS staining of kidneys from sham, 30min-IRI, and 33min-IRI group at day 1 after surgery. Scale bar, 25 μm. Arrows indicate damaged renal tubules

We established an ischemic renal injury mouse model by changing the clamping time of the bilateral renal pedicle to control the severity of the injury. The 7-day mortality in the 33min-IRI group reached 90% (Fig. 1a) (*n* = 21), and the blood creatinine level on the first day after surgery was 1.95 ± 0.15 mg/dl (Fig. 1b), which was in line with the characteristics of clinically severe AKI. There was no death in the 30min-IRI group (Fig. 1a), and the postoperative day 1 serum creatinine was 0.97 ± 0.3 mg/dl, which was corresponding to clinically moderate AKI (Fig. 1b). The mice subjected to renal IRI showed pronounced renal pathological damage compared to mice in the sham group as indicated by periodic acid-Schiff (PAS) staining (Fig. 1c). Therefore, we successfully established a mouse model of IRI-AKI. Due to the significant natural death of the 33min-IRI group, the severe IRI-AKI model was used to observe the 7-day mortality of mice, while the 30min-IRI group of moderate IRI-AKI was mainly used to assess mouse kidney damage and subsequent mechanism study.

**Fig. 2:**
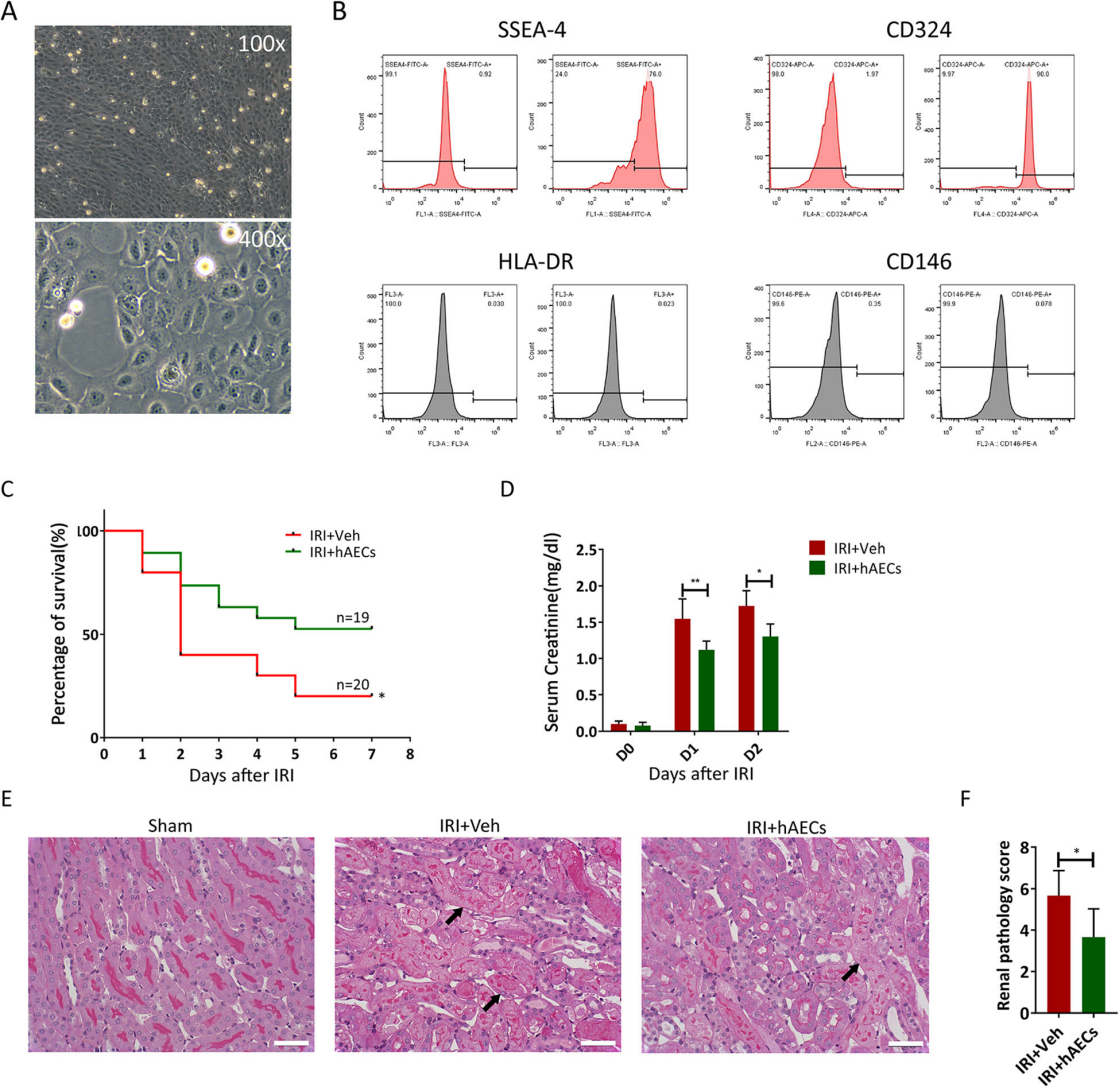
hAECs ameliorated acute renal IRI. **a** Morphology of hAECs was observed under bright field microscopy. Magnification, × 100 and × 400. **b** Flow cytometry analysis of cell surface markers SSEA4, CD324, HLA-DR, and CD146 on hAECs. The isotypes (ISO) were used as negative controls. hAECs are positive for SSEA4 and CD324 and negative for HLA-DR and CD146. **c** 7-day mortality rate in IRI mice with 33-min ischemia followed by vehicle (*n* = 19) or hAECs (*n* = 20) injection. *P < 0.05. **d** Serum creatinine concentrations in mice with 30 min ischemia followed by vehicle (*n* = 11) or hAECs injection (*n* = 13). *P < 0.05 vs IRI+Veh group, **P < 0.01 vs IRI+Veh group. **e** PAS staining of post-ischemic kidneys on day 1 after 30 min ischemia. Scale bar, 25 μm. Arrows indicate damaged renal tubules. **f** Renal pathological scores representing the degree of tubulointerstitial damage. *P < 0.05 vs IRI+Veh group. IRI+Veh: 30-min ischemia mice injected with vehicle alone (*n* = 3); IRI+hAECs: 30-min ischemia mice injected with 1 × 10^6^ hAECs (*n* = 3). Data are shown as mean (SD)

We next examined the effects of hAECs on IRI renal damage. The hAEC cells were characterized by checking their phenotypes under light microscopy (Fig. 2a) and detecting stem cell markers SSEA-4, CD324, HLA-DR, and CD146 by flow cytometry (Fig. 2b). Injection of hAECs reduced the 7-day mortality from 90 to 47.4% (*p* < 0.05) in the 33min-IRI severe AKI group (Fig. 2c). Serum creatinine levels were decreased in hAECs group on day 1 and day 2 post 30-min ischemia (Fig. 2d). The proportion of necrotic and damaged tubules tended to be lower in the cortex and outer medullar regions of the hAEC-treated group than in the vehicle control (Fig. 2e and f). These findings indicate that hAECs can promote renal function and enhance mice survival in IRI-AKI.

### hAECs-EXO recapitulated hAECs’ protective effect on IRI-AKI

**Fig. 3:**
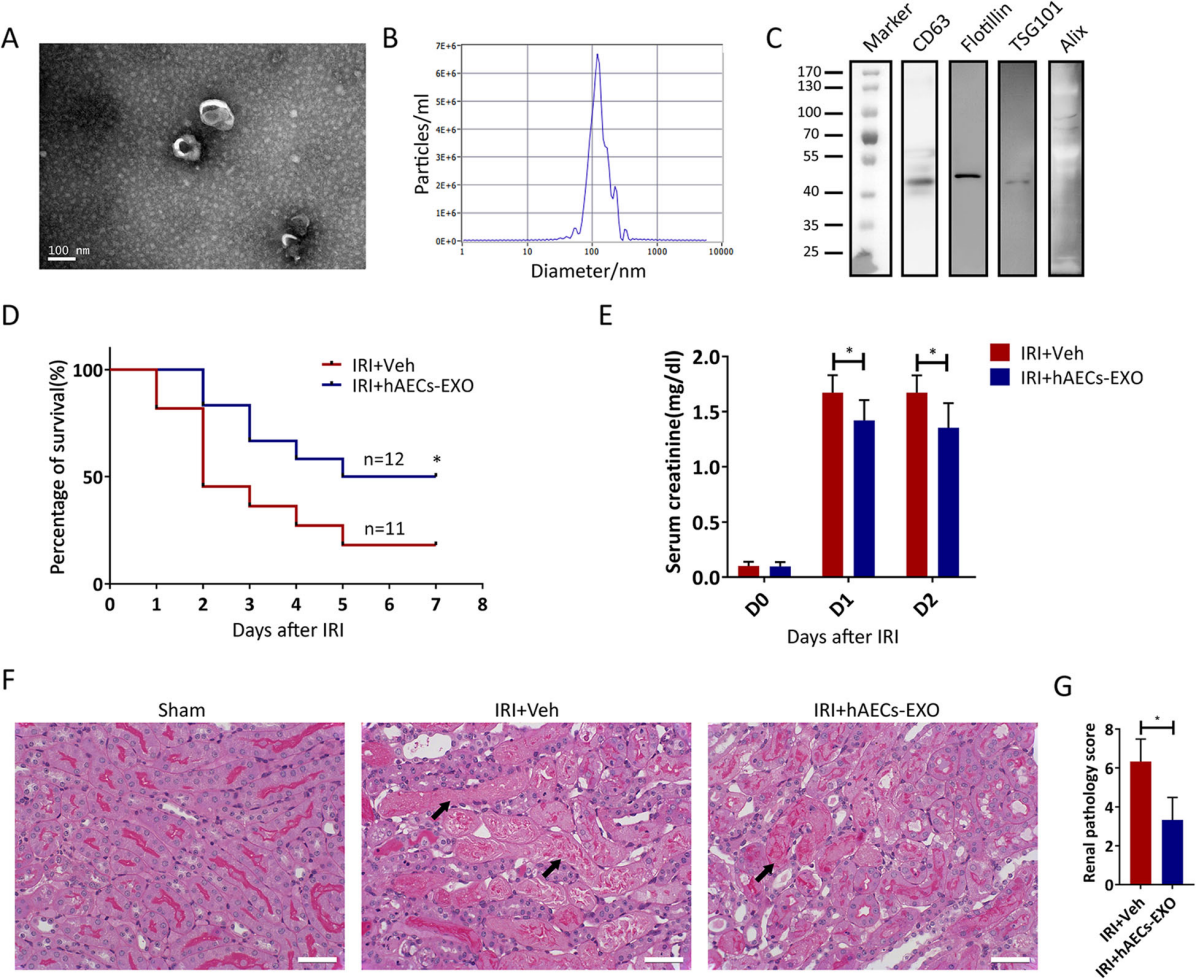
Effects of intravenous injection of hAECs-EXO in mice with IRI-AKI. **a** Morphology of hAECs exosomes under transmission electron microscopy. Scale bar, 100 nm. **b** Mean diameter and concentration of hAEC exosomes analyzed by nanoparticle tracking system (NTA). Approximately 1.6 × 10^10^ particles were measured by NTA in hAECs exosomes, which came from a total of 48.2 × 10^6^ hAECs. **c** Western blot detection of the exosome markers CD63, Flotillin, TSG101, and Alix. **d** 7-day mortality rate in mice with 33-min ischemia followed by vehicle (IRI+Veh, *n* = 11) or hAECs-EXO (IRI+hAECs-EXO, *n* = 12) injection. *P < 0.05. **e** Serum creatinine concentrations in mice with 30-min ischemia followed by vehicle (*n* = 5) or hAECs injection (*n* = 6). *P < 0.05 vs IRI+Veh group. **f** PAS staining of post-ischemic kidneys at day 1 after 30-min ischemia in different groups as indicated. Scale bar, 25 μm. Arrows indicate damaged renal tubules. **g** Renal pathological scores representing the degree of tubulointerstitial damage. *P < 0.05 vs IRI+Veh group. IRI+Veh: 30-min ischemia mice injected with vehicle alone; IRI+hAECs-EXO: 30-min ischemia mice injected with about 3 × 10^8^ exosomes. Data are shown as mean (SD)

We examined the organ engraftment of hAECs after transplantation and found very low kidney tissue integration of hAECs from the beginning of injection till 7 days post injection despite a positive therapeutic signal in the IRI-AKI mice (Supplementary Figure1). Except the direct repopulation to the injured tissues, stem cells also exerted their therapeutic effect by paracrine of extracellular vesicles such as exosomes [27]. We isolated exosomes from hAEC-conditioned medium and tested their effects on IRI-AKI. The morphological assessment revealed the typical cup-shaped morphology of exosomes as determined by transmission electron microscopy (Fig. 3a). Size analysis using a nanoparticle tracking system showed a peak size of 50–150 nm (Fig. 3b). Western blot results showed the presence of exosomal markers, including CD63, Flotillin, TSG101, and Alix in the hAEC exosomes (Fig. 3c).

As shown in Fig. 3d, injection of hAECs-EXO reduced the 7-day mortality from 81.8 to 50% (*p* < 0.05) in 33min-IRI group. In 30min-IRI group, serum creatinine levels were significantly decreased (*p* < 0.05 on post-surgery day 1 and day 2) (Fig. 3e) and renal tubule necrosis was markedly reversed after hAECs-EXO injection (Fig. 3f, g).

### hAECs and hAECs-EXO protected kidney from IRI-AKI by reducing apoptosis and stimulating cell proliferation

**Fig. 4:**
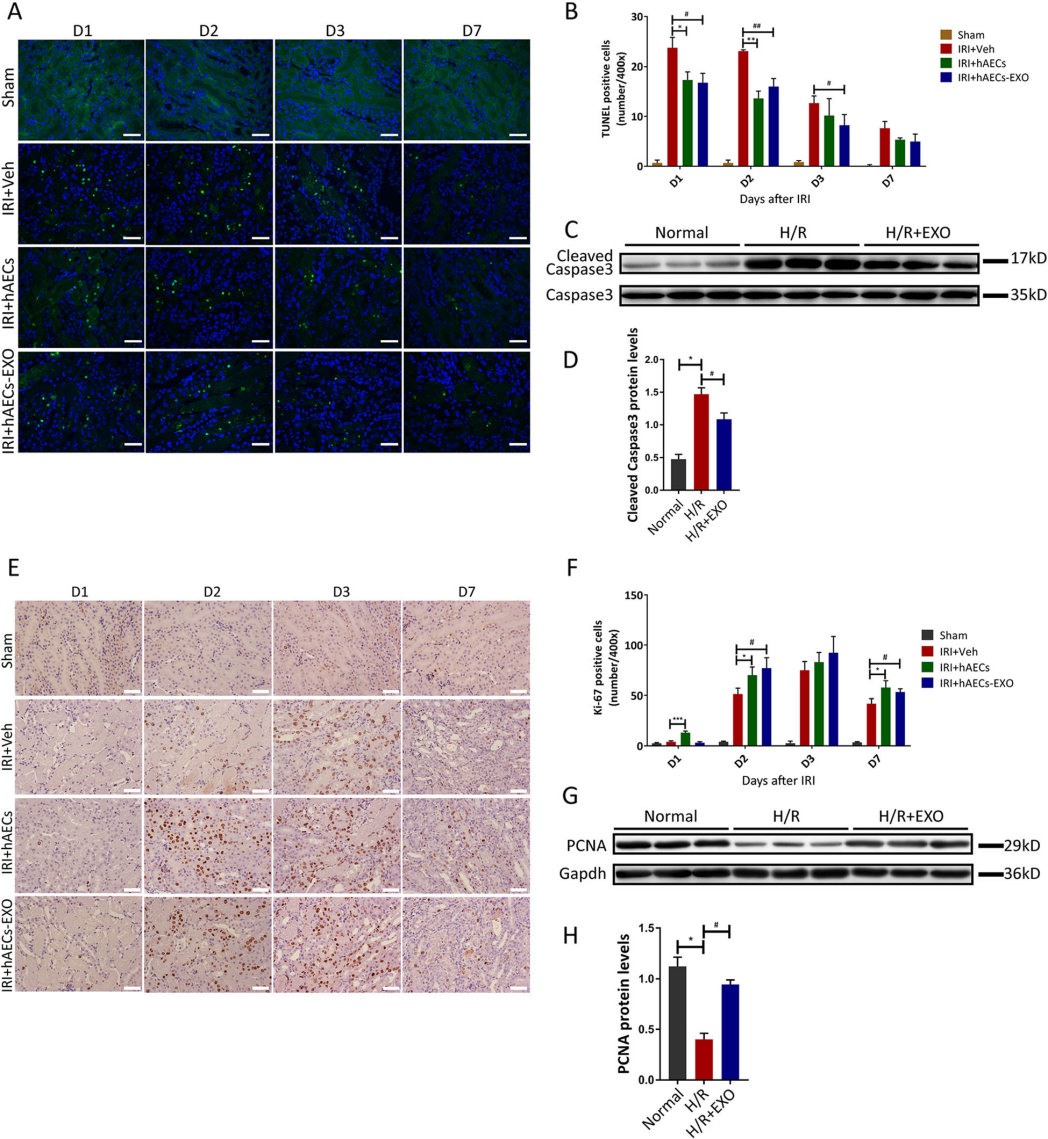
Anti-apoptotic effect of hAECs or hAECs-EXO in mice with 30-min ischemia and hAECs-EXO in H/R injured HK2 cells. **a** Representative micrographs of TUNEL staining in different groups as indicated at day 1, day 2, day 3, and day 7 post-ischemia. Scale Bar, 25 μm. **b** Quantification of TUNEL-positive cells/HPF. 3 different mice were used in each group with at least 6 images were taken on each mouse kidney. *P < 0.05 vs IRI+Veh group; **P < 0.01 vs IRI+Veh group; ^#^*P* < 0.05 vs IRI+Veh group; ^##^*P* < 0.01 vs IRI+Veh group. **c** Representative Western blots showed protein expression of cleaved-caspase 3 and caspase 3 in different groups as indicated. **d** Graphic presentation showed the relative abundances of cleaved-caspase 3 in different groups (*n* = 3). *P < 0.05 vs normal control; ^#^*P* < 0.05 vs H/R group. **e** Representative micrographs of Ki67 staining in different groups as indicated at day 1, day 2, day 3, and day 7 post-ischemia. Scale bar, 25 μm. **f** Quantification of Ki67-positive cells/HPF. 3 different mice were used in each group with at least 6 images were taken on each mouse kidney. *P < 0.05 vs IRI+Veh group; ***P < 0.001 vs IRI+Veh group; ^#^*P* < 0.05 vs IRI+Veh group. **g** Representative Western blots showed protein expression of PCNA in different groups as indicated (*n* = 3). **h** Graphic presentation showed the relative abundances of PCNA in different groups. *P < 0.05 vs normal control; ^#^*P* < 0.05 vs H/R group

On ischemic insults, tubular cells often undergo apoptosis or necrosis. When TUNEL-stained kidney tissues were observed by fluorescence microscopy, the number of apoptotic cells in the IRI group was significantly increased compared with that in the sham group (Fig. 4a). However, as shown in Fig. 4b, hAECs or hAECs-EXO treatment significantly decreased the number of apoptotic cells. Moreover, in vitro, HK-2 cells were cultured in a hypoxic environment to simulate ischemia within the tissue, and proteins from the HK-2 cells treated with or without hypoxia-reoxygenation (H/R) injury were collected. Western blot analysis showed that the expression level of apoptosis-related marker cleaved caspase-3 in the H/R group was significantly higher than that in the control group, while hAECs-EXO treatment significantly suppressed cleaved casapase-3 expression (Fig. 4c, d).

Meanwhile, hAECs and hAECs-EXO stimulated dramatic tubular cell proliferation 2 days after surgery as shown by anti-Ki67 staining (Fig. 4e, f). Similarly, in H/R-stimulated HK-2 cells, the expression of PCNA was significantly higher in hAECs-EXO-treated group than H/R group (Fig. 4g, h). Taken together, these observations demonstrated that hAEC exosomes enhanced cell proliferation and attenuated apoptosis in renal epithelial cells under H/R injury.

### hAECs and hAECs-EXO prevent peritubular capillary loss

**Fig. 5:**
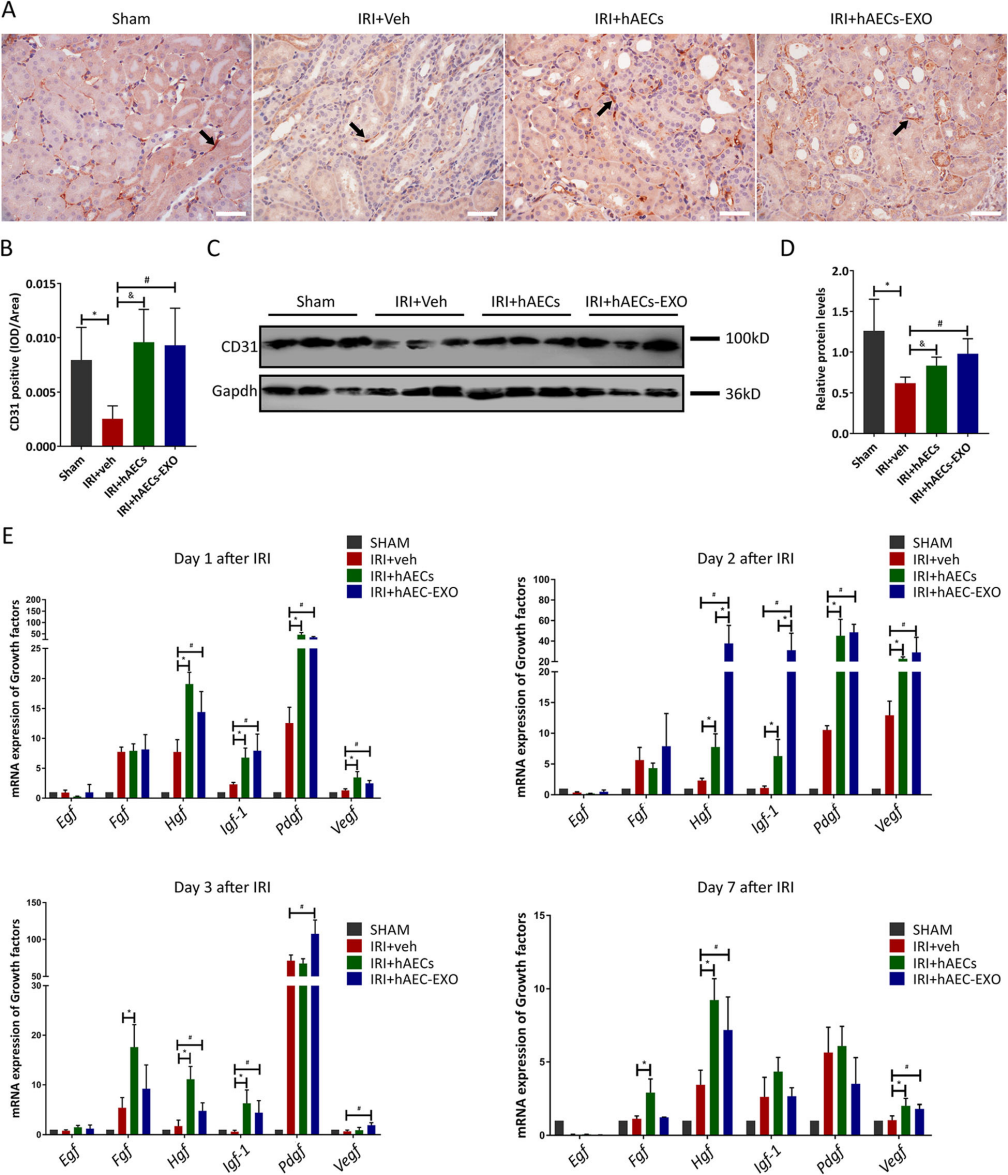
hAECs or hAECs-EXO prevent peritubular capillary loss in mice with 30-min ischemia. **a** Representative micrographs of CD31 staining in different groups as indicated at day 7 post-ischemia. Arrows indicate CD31 positive peritubular capillaries (PTCs). Scale bar, 25 μm. **b** Quantification of CD31-positive signal/HPF. 3 different mice were used in each group with at least 6 images were taken on each mouse kidney. *P < 0.05 vs sham group; ^&^*P* < 0.05 vs IRI+Veh group; ^#^*P* < 0.05 vs IRI+Veh group. **c** Representative Western blot analyses showed protein expression of CD31 in different groups as indicated. **d** Graphic presentation showed the relative abundances of CD31 in different groups. *P < 0.05 vs sham group; ^&^*P* < 0.05 vs IRI+Veh group; ^#^*P* < 0.05 vs IRI+Veh group (*n* = 3). **e** Growth factors expression in post-ischemic kidneys on day 1, day 2, day 3, and day 7 after hAECs or hAECs-EXO administration in mice with IRI-induced AKI. mRNA transcripts of *Egf*, *Fgf*, *Hgf*, *Igf-1*, *Pdgf*, and *Vegf* were determined by qRT-PCR. *P < 0.05 vs IRI+Veh group; ^#^*P* < 0.05 vs IRI+Veh group (*n* = 4)

The tubular injuries in IRI-AKI are accompanied by renal vascular impairment. We next investigated the change of peritubular capillary (PTC) density in IRI kidneys by immunohistochemistry and Western blot analysis using PTC endothelial cell marker, CD31. CD31+ capillaries were found to be intact in the sham kidney (Fig. 5a). Ischemia induced significant PTC loss in the kidneys on the 7th day of reperfusion (*p* < 0.05). However, the density of peritubular capillaries was highly increased by hAEC transplantation or hAECs-EXO administration (*p* < 0.05) post ischemic injury (Fig. 5a–d). We also determined the mRNA levels of the angiogenesis-related genes (*Egf*, *Fgf*, *Hgf*, *Igf-1*, *Pdgf*, and *Vegf*) in the IRI kidneys. The expression of these regeneration markers was very weak in sham kidneys, and IRI induced a slight increase in the gene expression. hAECs and hAECs-EXO treatment significantly upregulated the expression of these genes, with only no obvious difference in *Egf* expression between vehicle control group and hAECs or hAECs-EXO treatment groups (Fig. 5e).

### hAECs and hAECs-EXO increased M2 macrophage polarization with a concomitant modulation of inflammatory cytokines

**Fig. 6:**
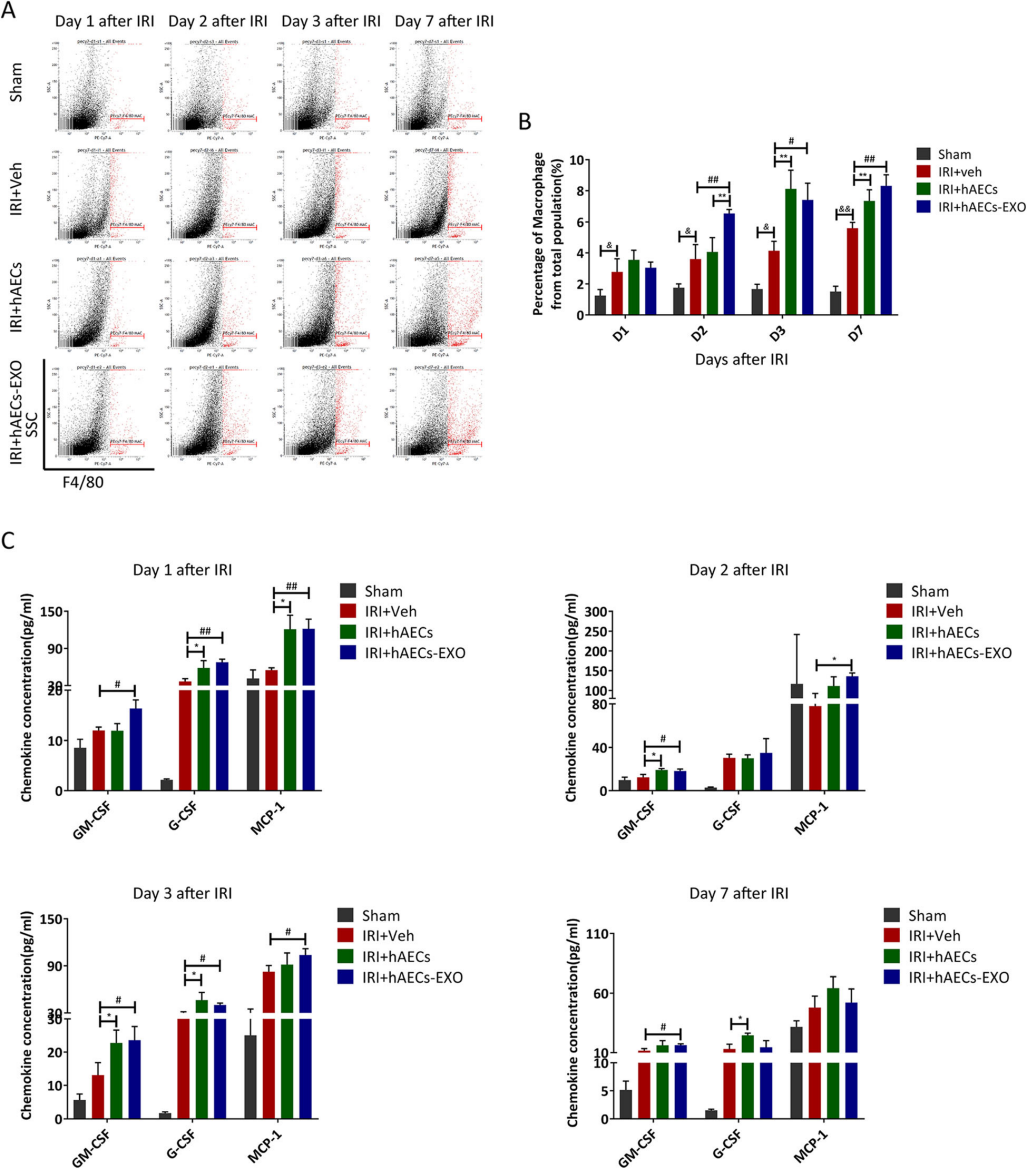
hAECs altered chemokine expression and macrophage infiltration in kidneys with 30-min ischemia. **a** Macrophage populations were measured via flow cytometry. Representative gating strategy was shown. The percentages of macrophages from the total kidney cell population were calculated. **b** Percentage of F4/80+ macrophages in kidneys treated with hAECs or hAECs-EXO at day 1, day 2, day 3, and day 7 post-ischemia. ^&^*P* < 0.05 vs sham group; ^&&^*P* < 0.01 vs sham group; **P < 0.01 vs IRI+Veh group; ^#^*P* < 0.05 vs IRI+Veh group; ^##^*P* < 0.01 vs IRI+Veh group. **c** Kidney chemokine concentrations from mice in different groups as indicated at day 1, day2, day 3, and day 7after IRI. *P < 0.05 vs IRI+Veh group; ^#^*P* < 0.05 vs IRI+Veh group; ^##^*P* < 0.01 vs IRI+Veh group (*n* = 3)

Stem cell therapy has been reported to prevent renal injuries via immune regulation [28, 29]. Macrophages are important immune cells with high heterogeneity and plasticity, and the imbalance of M1/M2 macrophages phenotypes contributes to the inflammation or tissue repair in acute kidney injury [30]. Ischemia reperfusion promoted macrophages infiltration into the kidney as shown by an increased percentage of macrophages after I/R compared to the sham group (Fig. 6a, b). A further increase of macrophage population was observed during the 7-day course of reperfusion in hAEC- and hAECs-EXO-treated kidney (Fig. 6a, b), in consistent with the high levels of macrophage attractive chemokines in local kidney (Fig. 6c).

**Fig. 7:**
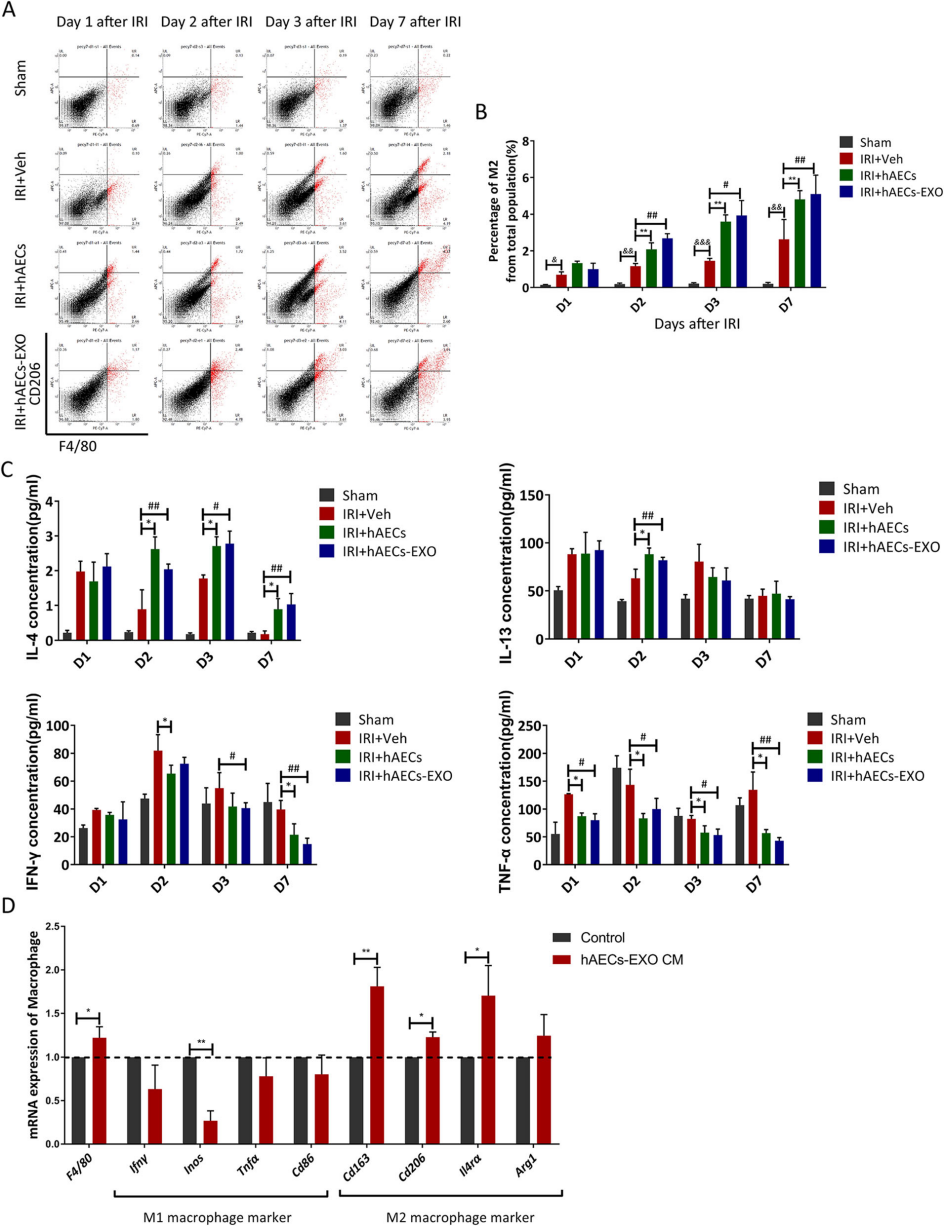
hAECs-EXO induced M2 macrophage polarization. **a** CD206+/F4/80+ M2 macrophage population was measured via flow cytometry. Representative gating strategy was shown. The percentages of M2 macrophages from the total kidney cell population were calculated. **b** Percentage of M2 Macrophages in kidneys treated with hAECs or hAECs-EXO at day 1, day 2, day 3, and day 7 post-ischemia (*n* = 3). ^&^*P* < 0.05 vs sham group; ^&&^*P* < 0.01 vs sham group; ^&&&^*P* < 0.001 vs sham group; **P < 0.01 vs IRI+Veh group. ^#^*P* < 0.05 vs IRI+Veh group; ^##^*P* < 0.01 vs IRI+Veh group. **c** Kidney cytokine concentrations from mice in different groups as indicated at day 1, day2, day 3, and day 7 after IRI (*n* = 3). *P < 0.05 vs IRI+Veh group; ^#^*P* < 0.05 vs IRI+Veh group; ^##^*P* < 0.01 vs IRI+Veh group. **d** Bone marrow monocytes were attached for 48 h and collected as control. Bone marrow-derived macrophages were cultured in hAECs-EXO conditioned medium for 7 days and collected. mRNA transcripts of macrophage marker (*F4/80*) and M1 (*Ifn*γ, *iNos*, *Tnf*α, *Cd86*) and M2 (*Cd163*, *Cd206*, *Il4r*α, *Arg*1) markers were determined by qRT-PCR (*n* = 3). *P < 0.05 vs control group; **P < 0.01 vs control group. Data are shown as mean (SD)

The phenotype of local macrophages was determined by flow cytometry (Fig. 7a). I/R animals exhibited prominent differentiation of CD206+/F4/80+ M2 type macrophages in the kidneys on day 2 and afterwards after hAECs transplantation or hAECs-EXO administration (Fig. 7b). Concomitantly, post-ischemic kidneys of the hAEC or hAECs-EXO-treated groups expressed higher levels of anti-inflammatory cytokines such as IL4 and IL13, and lower levels of proinflammatory cytokines such as IFNγ and TNFα, than those of the vehicle-treated group (Fig. 7c). In vitro, using hAECs-EXO as supplement to culture bone marrow-derived macrophages for 7 days, the gene expression of M2 markers including *Cd206*, *Cd163*, *Il4r*α, and *Arg*1 was significantly increased, with a downregulation of gene expression of M1 markers such as *Cd86*, *Ifn*γ, *Tnf*α, and *iNos* (Fig. 7d). Thus, we postulated that hAECs could alleviate IRI-AKI probably through promoting M2 macrophage polarization and inhibiting the systemic inflammation.

### Proteomic profiling of hAECs-EXO

Above data revealed that hAEC-derived exosomes displayed renal protective functions similar to parent cells, indicating the paracrine pathway played an important role in the process of hAEC-mediated kidney tissue functional recovery. A comprehensive analysis of the proteome on hAECs-EXO allowed an overall identification of 171 proteins (Supplementary Table 2). The functional annotation of these proteins was performed using DAVID functional enrichment analysis tool. Predictably, a large proportion of exosome proteins was annotated as either of extracellular or membrane origin, with a sizable contribution to the cytosol and ER lumen (Supplementary Figure 2A). The molecular functions of these exosome proteins were mainly involved in protein binding, integrin binding, heparin binding, cadherin binding, and extracellular matrix structural constituent (Supplementary Figure 2B). The biological process category showed “extracellular matrix organization,” “cell adhesion,” “regulation of complement activation,” “leukocyte migration,” “innate immune response,” as well as “angiogenesis” and “positive regulation of cell proliferation” as the most enriched terms (Supplementary Figure 2C).

We next examined the detailed function of these 171 hAEC exosome proteins in Uniprot protein bank (http://www.uniprot.org) and found a large population of proteins were extracellular matrix (ECM) constituents (FN1, COL5A1, COL1A2, LAMA3, COL12A1, LAMB3, LAMC2, COL3A1, LAMC1, COL1A1, LAMB1, FBN1, COL7A1, VTN, LAMA5, COL6A1, FBLN1, LAMB2, FBN2, COL6A3) or Matricellular Proteins (TNXB, THBS1, THBS3, and OLFML3). We also found a series of proteins were involved in proliferation/apoptosis and angiogenesis. These signaling pathways included integrin signaling (ITGB3, ITGB6, ITGAV, ITGA6, ITGA2), IGF signaling (IGFBP2, IGF2), HGF signaling (HGFAC), TGFβ signaling (TGFBI, LTBP1, BMP1), Wnt signaling (DKK3, SFRP1, LRP1), and programmed cell death pathway (PDCD6IP). SPARC, SERPINE1, NID2, MYLK, CCDC80, TIMP1, WDR1, and the complement components (C3, C6, C4B, C1R, C1QB, C8B, C2, C5) were reported to participate in cell migration, cytokine production, and inflammation. Taken together, hAECs-EXO might serve as important carriers with anti-apoptotic, pro-angiogenic, and immunomodulatory proteins that could target multiple aspects of the IRI damage and ameliorate the acute kidney tissue injury.

## Discussion

Acute kidney injury is a clinical syndrome with a rapid decline in kidney function that ultimately increases mortality and morbidity. Despite the ever-increasing prevalence of acute kidney disease, there is a lack of potential therapeutic agents, leaving us with the option of costlier replacement therapies. In recent years, stem cell therapy has drawn much attention for the treatment of AKI [7]. As a main player, MSCs derived from different sources like adipose, bone-marrow and human umbilical cord have shown renoprotective effects in several models of toxin or IRI-induced AKI [31–33]. Meanwhile, numerous controversies have arisen regarding safety, cost, availability, and ethical considerations of MSCs [34]. In seeking of alternative cell sources, human amniotic epithelial cell is one promising option.

In the current study, we observed a dramatic decrease of mortality after hAECs treatment in severe IRI-AKI mice with 33-min ischemia, indicating the powerful protective potential of hAECs on ischemic injury. IRI-AKI has been associated with accumulation of reactive oxygen species (ROS) and apoptosis, loss of peritubular capillaries, and migration of pro-inflammatory immune cells to the damage tissue [2, 4]. In our study, we demonstrated that hAEC therapy could effectively reduce kidney injury in moderate IRI-AKI animal with 30-min ischemia. Intravenous injection of hAECs attenuated tubular cell apoptosis and necrosis and promoted cell proliferation in the injured kidney. Peritubular capillary rarefaction is a typical feature and important mechanism of the transition from an AKI insult to the development of chronic kidney fibrosis. The methods that restore the renal microvasculature have been regarded as a promising strategy to prevent AKI from progression to chronic kidney disease [35]. After hAEC administration, we observed a dramatic increase of the peritubular capillary density and upregulation of angiogenetic genes such as *Vegf* and *Pdgf* in the injured kidney. hAECs also reprogramed macrophages to shift from a pro-inflammatory M1 to anti-inflammatory M2 state. This shift was associated with the increased levels of IL4 and IL13 and decreased levels of TNFα and IFNγ, which in turn helped to reduce the inflammatory response. Taken together, these anti-inflammatory, anti-apoptotic, and pro-angiogenetic effects of hAECs were associated with both the rapid recovery of kidney function and the enhanced survival in the mice with IRI kidney injury.

It was originally believed that stem cells provided a therapeutic basis for regeneration by engrafting at the site of injury via transdifferentiation. However, further investigation revealed that engraftment of stem cells to the site of injury was very rare and therefore was not likely to be the sole cause of regeneration after AKI [36]. In our case, we found very few kidney tissue integration of hAECs after transplantation. The majority of hAECs were concentrated in the lung and heart. Growing evidence in regenerative medicine supports the hypothesis that stem cells exert their therapeutic effect by a paracrine manner rather than a direct repopulation to the injured tissues [27]. Exosomes secreted from stem cells could act as major transporters in cell-cell communication to deliver bioactive molecules from original cells to the recipient cells [37]. It has been reported that hAEC-derived exosomes can restore ovarian function in chemotherapy-induced premature ovarian failure by transferring microRNAs against apoptosis [38]. In our study, we found that injection of exosomes derived from hAECs recapitulated hAECs’ protective effects, limiting apoptosis, enhancing proliferation, preventing peritubular capillary loss, and regulating immune response, thus reducing ischemia-reperfusion induced kidney damage.

We characterized the molecular compositions of hAECs-EXO by proteomic approach to find out the specific mechanisms of hAEC-based therapy in AKI. Cellular processes including “extracellular matrix organization,” “cell adhesion,” “leukocyte migration” as well as “angiogenesis” and “positive regulation of cell proliferation” were enriched in hAECs-EXO. Proteins involved in the IGF signaling, HIF signaling, integrin signaling, Wnt signaling, and TGFβ signaling were detected in hAECs-EXO. ECM proteins are also abundant in hAECs-EXO. It has been shown that ECM processing and cell-ECM interactions are major determinants in the regulation of signaling pathways that drive kidney repair [39, 40]. In addition to the classical ECM structural proteins, we found that several matricellular proteins (MCPs) such as TNXB, THBS1, and THBS3 were detected in hAECs-EXO with high abundance. MCPs are ECM-bound nonstructural proteins that interact with integrins, growth factor receptors, and growth factors to modulate their function and activity [41, 42]. In our proteomic analysis, Tenascin X (TNXB) as a typical matricellular protein stands out. An in vitro study has shown that TNX physically interacts with VEGF-B and enhances the ability of VEGF-B to stimulate endothelial cell proliferation [43], suggesting a role for TNXB in the regulation of angiogenesis. It was found that in normal kidney tenascin expression was limited to the medullary interstitium [44]. Chen et al. reported that Tenascin-C (TNC), a member of the tenascin family, was specifically induced at sites of injury and recruited Wnt ligands, thereby creating a favorable microenvironment for tubular repair and regeneration after ischemia reperfusion-induced AKI [45]. Thus, it would be interesting to explore the in vivo role of TNXB on kidney regeneration in IRI models, leading to an elucidation of the molecular mechanism of hAECs therapy for IRI-AKI.

## Conclusion

In summary, the above findings advance our knowledge of the therapeutic potential of hAECs in ischemia reperfusion-induced AKI mouse model, pointing to hAECs as an attractive candidate for ameliorating renal tissue damage, possibly through the limitation of apoptosis, prevention of capillary rarefraction and immunomodulation. Our data also demonstrate the renoprotective effects of hAEC-derived exosomes, which overcomes the weaknesses and risks associated with the use of native stem cells and therefore could be a promising clinical therapeutic tool for patients with ischemic AKI.

## Supplementary information

**Additional file 1: Supplementary methods.** 1. Human mitocondria DNA detection in IRI mice injected with hAECs. 2. Immunofluorescence staining for humannuclear antigen (HNA). 3. Proteomics analysis.

**Additional file 2: Supplementary Figure 1.** hAECs mouse organ distribution. A. 100 ng total DNA was used as template and human mitochondria specific sequence was amplified. PCR products was detected by 1% agarose gel electrophoresis. hAECs was mainly concentrated in the lung and heart, much less hAECs DNA was detected in the kidney. B. Human nuclear antigen (HNA) staining for hAECs in lung and kidney tissue at 1 h and 1 day after tail injection of 1 × 10^6^ hAECs into the IRI mice. Green boxes indicate the HNA positive hAEC cells. Scale bar: 25 μm.

**Additional file 3: Supplementary Figure 2.** Proteomic profile of exosomes derived from hAECs. A and B, Gene ontology (GO) enrichment analysis for the significantly enriched GO terms of Cellular Component and Molecular Function. C, Bubble chart of the biological processes significantly enriched in hAECs exosome.

**Additional file 4: Supplementary Table 1.** Human amniotic epithelial cells ameliorate kidneydamage in ischemia-reperfusion mouse model of acute kidney injury.

**Additional file 5: Supplementary Table 2.** Proteins in hAECs-EXO identified by label-free shotgun proteomics approach.

## Data Availability

The datasets used and/or analyzed during the current study are available from the corresponding author on reasonable request.

## Abbreviations

AKI: Acute kidney injury
ROS: Reactive oxygen species
hAECs: Human amnion epithelial cells
hAECs-EXO: Human amnion epithelial cell-derived exosomes
I/R: Ischemia reperfusion
IRI: Ischemic-reperfusion injury
IRI-AKI: Ischemic-reperfusion injury-induced acute kidney injury
H/R: Hypoxia-reoxygenation
EVs: Extracellular vesicles
NTA: Nanoparticle tracking analysis
PAS: Periodic acid-Schiff
FBS: Fetal bovine serum
PCNA: Proliferating cell nuclear antigen
PTC: Peritubular capillary
ECM: Extracellular matrix
MCP: Matricellular protein
TNXB: Tenascin-X
TNC: Tenascin-C

## References

[1] Bellomo R, Kellum JA, Ronco C (2012). Acute kidney injury. Lancet.

[2] Sharfuddin AA, Molitoris BA (2011). Pathophysiology of ischemic acute kidney injury. Nat Rev Nephrol.

[3] Bonventre JV, Yang L (2011). Cellular pathophysiology of ischemic acute kidney injury. J Clin Invest.

[4] Zuk A, Bonventre JV (2016). Acute kidney injury. Annu Rev Med.

[5] Molitoris BA (2014). Therapeutic translation in acute kidney injury: the epithelial/endothelial axis. J Clin Invest.

[6] Chatterjee PK (2007). Novel pharmacological approaches to the treatment of renal ischemia-reperfusion injury: a comprehensive review. Naunyn Schmiedeberg’s Arch Pharmacol.

[7] Barnes CJ, Distaso CT, Spitz KM (2016). Comparison of stem cell therapies for acute kidney injury. Am J Stem Cells.

[8] Miki T (2018). Stem cell characteristics and the therapeutic potential of amniotic epithelial cells. Am J Reprod Immunol.

[9] Cargnoni A, Piccinelli EC, Ressel L (2014). Conditioned medium from amniotic membrane-derived cells prevents lung fibrosis and preserves blood gas exchanges in bleomycin-injured mice-specificity of the effects and insights into possible mechanisms. Cytotherapy.

[10] Kakishita K, Elwan MA, Nakao N (2000). Human amniotic epithelial cells produce dopamine and survive after implantation into the striatum of a rat model of Parkinson's disease: a potential source of donor for transplantation therapy. Exp Neurol.

[11] Manuelpillai U, Tchongue J, Lourensz D (2010). Transplantation of human amnion epithelial cells reduces hepatic fibrosis in immunocompetent CCl4-treated mice [in English]. Cell Transplant.

[12] Toegel F, Hu ZM, Weiss K (2005). Administered Mesenchymal stem cells protect against ischemic acute renal failure through paracrine and anti-inflammatory mechanisms [in English]. Nephrology.

[13] Duffield JS, Park KM, Hsiao LL (2005). Restoration of tubular epithelial cells during repair of the postischemic kidney occurs independently of bone marrow-derived stem cells [in English]. J Clin Invest.

[14] Heijnen HF, Schiel AE, Fijnheer R (1999). Activated platelets release two types of membrane vesicles: microvesicles by surface shedding and exosomes derived from exocytosis of multivesicular bodies and alpha-granules. Blood.

[15] Record M, Silvente-Poirot S, Poirot M (2018). Extracellular vesicles: lipids as key components of their biogenesis and functions. J Lipid Res.

[16] Keerthikumar S, Chisanga D, Ariyaratne D (2016). ExoCarta: a web-based compendium of exosomal cargo [in English]. J Mol Biol.

[17] Alhomrani M, Correia J, Hodge A (2016). Extracellular vesicles derived from human amnion epithelial cells reduce liver fibrosis through effects on hepatic stellate cells and macrophages [in English]. Hepatology.

[18] Murphy S, Lim R, Dickinson H (2011). Human amnion epithelial cells prevent Bleomycin-induced lung injury and preserve lung function [in English]. Cell Transplant.

[19] Gurunathan S, Kang MH, Jeyaraj M et al. Review of the isolation, characterization, biological function, and multifarious therapeutic approaches of exosomes [in English]. Cells-Basel 2019;8(4):307-43. 10.3390/cells8040307 PMC6523673 30987213

[20] Gregorini M, Corradetti V, Pattonieri EF (2017). Perfusion of isolated rat kidney with mesenchymal stromal cells/extracellular vesicles prevents ischaemic injury. J Cell Mol Med.

[21] Cantaluppi V, Gatti S, Medica D (2012). Microvesicles derived from endothelial progenitor cells protect the kidney from ischemia-reperfusion injury by microRNA-dependent reprogramming of resident renal cells [in English]. Kidney Int.

[22] Wei Q, Dong Z (2012). Mouse model of ischemic acute kidney injury: technical notes and tricks. Am J Physiol Renal Physiol.

[23] Jiang HR, Park JH, Kwon GY, et al. Effect of preemptive treatment with human umbilical cord blood-derived mesenchymal stem cells on thedevelopment of renal ischemia-reperfusion injury in mice. Am J Physiol Renal Physiol. 2014;307(10):F1149-1161. 10.1152/ajprenal.00555.2013 25143451

[24] Torres Crigna A, Daniele C, Gamez C (2018). Stem/stromal cells for treatment of kidney injuries with focus on preclinical models. Front Med (Lausanne).

[25] Zahedi K, Revelo MP, Barone S (2006). Stathmin-deficient mice develop fibrosis and show delayed recovery from ischemic-reperfusion injury [in English]. Am J Physiol-Renal.

[26] Manzanero S (2012). Generation of mouse bone marrow-derived macrophages. Methods Mol Biol.

[27] Grange C, Skovronova R, Marabese F et al. Stem cell-derived extracellular vesicles and kidney regeneration. Cells. 2019;8(10):1240-53. 10.3390/cells8101240 PMC6830104 31614642

[28] Tseng W, Hung S, Chien C (2018). Hypoxia-preconditioned mesenchymal stem cells mitigate renal ischemia-reperfusion injury by modulating M2 macrophage subsets and suppressing oxidative stress [in English]. Cytotherapy.

[29] Yuan YJ, Li L, Zhu LL, et al. Mesenchymal stem cells elicit macrophages into M2 phenotype via improving transcription factor EB-mediated autophagy to alleviate diabetic nephropathy [in English]. Stem Cells. 2020;38(5):639-52. 10.1002/stem.3144 31904160

[30] Huen SC, Cantley LG (2017). Macrophages in renal injury and repair. Annu Rev Physiol.

[31] Zhang JB, Wang XQ, Lu GL (2017). Adipose-derived mesenchymal stem cells therapy for acute kidney injury induced by ischemia-reperfusion in a rat model. Clin Exp Pharmacol Physiol.

[32] Geng Y, Zhang L, Fu B (2014). Mesenchymal stem cells ameliorate rhabdomyolysis-induced acute kidney injury via the activation of M2 macrophages. Stem Cell Res Ther.

[33] Fang TC, Pang CY, Chiu SC (2012). Renoprotective effect of human umbilical cord-derived mesenchymal stem cells in immunodeficient mice suffering from acute kidney injury. PLoS One.

[34] Musial-Wysocka A, Kot M, Majka M (2019). The pros and cons of mesenchymal stem cell-based therapies [in English]. Cell Transplant.

[35] Long DA, Norman JT, Fine LG (2012). Restoring the renal microvasculature to treat chronic kidney disease [in English]. Nat Rev Nephrol.

[36] Phinney DG, Prockop DJ (2007). Concise review: mesenchymal stem/multipotent stromal cells: the state of transdifferentiation and modes of tissue repair--current views. Stem Cells.

[37] Hur YH, Cerione RA, Antonyak MA. Extracellular vesicles and their roles in stem cell biology [in English]. Stem Cells. 2020;38(4):469-76. 10.1002/stem.3140 PMC7703835 31828924

[38] Zhang Q, Sun J, Huang Y (2019). Human amniotic epithelial cell-derived exosomes restore ovarian function by transferring microRNAs against apoptosis. Mol Ther Nucleic Acids.

[39] Wynn TA, Ramalingam TR (2012). Mechanisms of fibrosis: therapeutic translation for fibrotic disease [in English]. Nat Med.

[40] Liu YH (2006). Renal fibrosis: new insights into the pathogenesis and therapeutics [in English]. Kidney Int.

[41] Bornstein P (2009). Matricellular proteins: an overview [in English]. J Cell Commun Signal.

[42] Murphy-Ullrich JE, Sage EH (2014). Revisiting the matricellular concept [in English]. Matrix Biol.

[43] Ikuta T, Ariga H, Matsumoto K (2000). Extracellular matrix tenascin-X in combination with vascular endothelial growth factor B enhances endothelial cell proliferation [in English]. Genes Cells.

[44] Truong LD, Foster SV, Barrios R (1996). Tenascin is an ubiquitous extracellular matrix protein of human renal interstitium in normal and pathologic conditions [in English]. Nephron.

[45] Chen SQ, Fu HY, Wu SZ (2019). Tenascin-C protects against acute kidney injury by recruiting Wnt ligands [in English]. Kidney Int.

