# Supplementary material for: Human amniotic epithelial cells ameliorate kidney damage in ischemia-reperfusion mouse model of acute kidney injury
Source: Stem Cell Res Ther. 2020 Sep 23;11:410. doi: 10.1186/s13287-020-01917-y (PMC7510147; doi:10.1186/s13287-020-01917-y)
Supplement: Supplementary file 1 — Additional file 1: Supplementary methods. 1. Human mitocondria DNA detection in IRI mice injected with hAECs. 2. Immunofluorescence staining for humannuclear antigen (HNA). 3. Proteomics analysis. [file 13287_2020_1917_MOESM1_ESM.docx]

**Supplementary methods**

Human mitochondria DNA detection in IRI mice injected with hAECs

1×10^6^ hAECs were injected into the IRI mouse tail vein immediately after surgery. Heart, liver, spleen, lung and kidney tissues were collected 1 hour, 1 day, 2 days, 3 days and 7 days after injection. Tissue DNA were extracted by mammalian genomic DNA extraction kit (Beyotime, D0061, Beijing, China). Total DNA concentration was detected by NanoDrop 2000 Spectrophotometer. 100ng DNA was used as PCR template for each organ. Human mitochondria DNA specific primer sequences were hmtDNA-FP:5′CAC CCT ATT AAC CAC TCA CG 3′; hmtDNA-RP: 5′-ATG TCT GTG TGG AAA GCG-3′. PCR program was as following: 94℃ 3 min, 94℃ denaturation for 30 s, 55℃ annealing for 30 s, 72℃ extension for 30, with 40 cycles. The product size was 264 bp.

Immunofluorescence staining for human nuclear antigen (HNA)

Human nuclear antigen (HNA) was stained on frozen sections to locate the engrafted hAECs in the IRI mouse organ. Kidney and lung tissues were collected 1 hour and 1 day after tail vein injection of 1×10^6^ hAECs into the mice. The primary antibody was mouse anti-HNA (1:100, Cat. No.191181, Abcam). The slides were then exposed to cy3-conjugated secondary antibody. DAPI was counter stained to show nuclear. The staining was examined using Nikon 90i microscope (Nikon, Japan).

Proteomics analysis

Peptide preparation for proteomic analysis was performed as described previously. Briefly, 300ug exosome proteins isolated from hAECs were used to perform the analysis with triplicates. Proteins were separated using 4–15% Mini-PROTEAN® TGXTM Precast Protein Gel (BioRad, Hercules, CA, USA), stained with Coomassie Brilliant Blue R-250 staining solution (BioRad, Hercules, CA, USA). Total proteins resolved using mono-dimensional gel electrophoresis was subjected to protein-in-gel digestion. The peptides of each sample were desalted by a C18 Cartridge, concentrated by vacuum centrifugation and reconstituted in 40 μL of 0.1% formic acid.

The MS experiments were performed on a Q-Exactive HF Hybrid quadrupole-Orbitrap mass spectrometer (Thermo Fisher Scientific, Hemel Hempstead, UK) coupled to an Easy nLC nano-liquid chromatography (Thermo Fisher Scientific, Hemel Hempstead, UK). The desalted peptides were separated on a 15cm long separation column with an inner diameter of 50 um (Acckaim PepMap RSLC, nano viper, P/N164943, Thermo Fisher Scientific, USA) using gradient of buffer A (Ultrapure water, 0.1% formic acid) and buffer B (Acetonitrile, 0.1% formic acid) at a flowrate of 300 nL min-1. The chromatographic gradient was set to provide a linear increase from 3% to 80% buffer B in 110 min, for a total run time of 120 min. MS data was acquired on data dependent mode dynamically choosing the top ten most abundant precursor ions from the survey scan (350-1800 m/z) for fragmentation and MS/MS analysis. Precursors with a charged state of +1 were rejected and the dynamic exclusion duration was set as 25 s. MS raw data were processed using MaxQuant software version 1.5.5.1 according to the standard workflow with the built-in search engine Andromeda. Proteins were identified by searching against the Uniprot human reference proteome (Uniprot_HomoSapiens_20386_20180905) database. Carbamidomethylation of cysteines was set as fixed modification, while protein N-terminal acetylation and methionine oxidation were defined as variable modifications for peptide search. The false discovery rates (FDR) for peptide and protein identifications were set to 0.01%. A maximum of two missed cleavages were allowed for tryptic digestion. The MaxLFQ label-free quantitation method [22] with retention time alignment and match-between-runs feature in MaxQuant was applied to extract the maximum possible quantification information. Protein abundance was calculated based on normalized spectral intensity (LFQ intensity).

The gene ontology (GO) annotation in categories such as cellular component, biological process, molecular functions of the significantly expressed proteins was carried out using DAVID functional enrichment analysis tool (https://david.ncifcrf.gov/).
