# Supplementary figures and images for: Human amniotic epithelial cells ameliorate kidney damage in ischemia-reperfusion mouse model of acute kidney injury
Source: Stem Cell Res Ther. 2020 Sep 23;11:410. doi: 10.1186/s13287-020-01917-y (PMC7510147; doi:10.1186/s13287-020-01917-y)

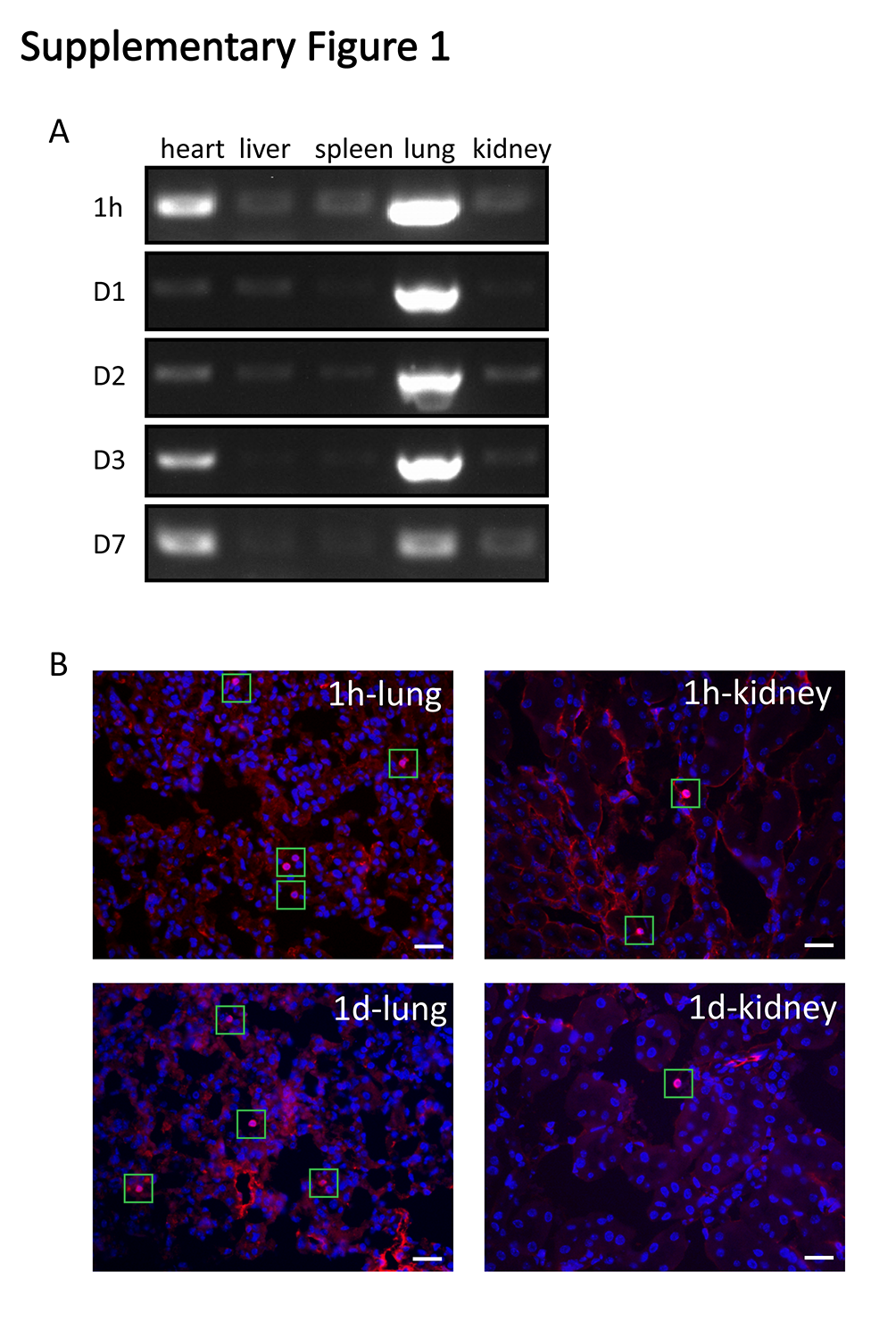

Supplement: Supplementary file 2 — Additional file 2: Supplementary Figure 1. hAECs mouse organ distribution. A. 100 ng total DNA was used as template and human mitochondria specific sequence was amplified. PCR products was detected by 1% agarose gel electrophoresis. hAECs was mainly concentrated in the lung and heart, much less hAECs DNA was detected in the kidney. B. Human nuclear antigen (HNA) staining for hAECs in lung and kidney tissue at 1 h and 1 day after tail injection of 1 × 10^6^ hAECs into the IRI mice. Green boxes indicate the HNA positive hAEC cells. Scale bar: 25 μm. [file 13287_2020_1917_MOESM2_ESM.tif]

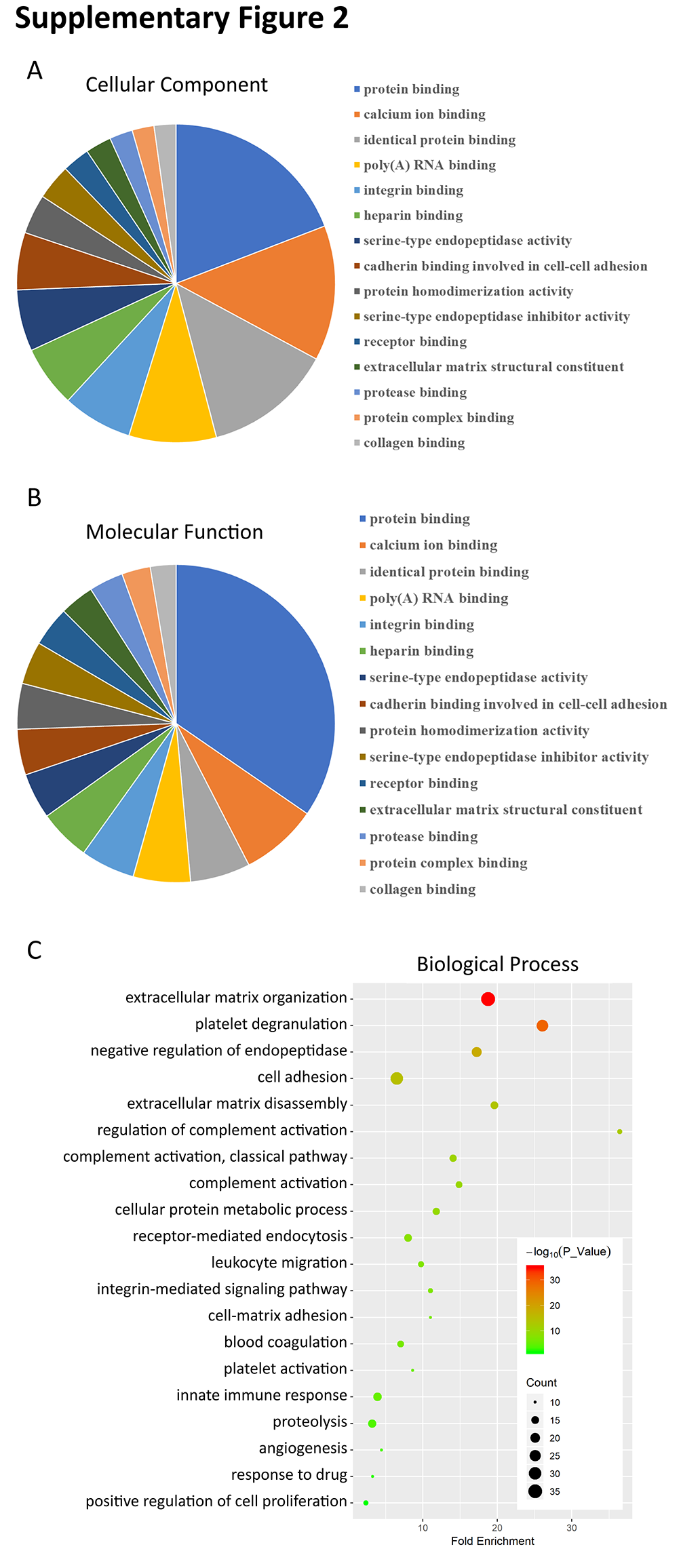

Supplement: Supplementary file 3 — Additional file 3: Supplementary Figure 2. Proteomic profile of exosomes derived from hAECs. A and B, Gene ontology (GO) enrichment analysis for the significantly enriched GO terms of Cellular Component and Molecular Function. C, Bubble chart of the biological processes significantly enriched in hAECs exosome. [file 13287_2020_1917_MOESM3_ESM.tif]
