# Supplementary material for: Human amniotic epithelial cells ameliorate kidney damage in ischemia-reperfusion mouse model of acute kidney injury
Source: Stem Cell Res Ther. 2020 Sep 23;11:410. doi: 10.1186/s13287-020-01917-y (PMC7510147; doi:10.1186/s13287-020-01917-y)
Supplement: Supplementary file 4 — Additional file 4: Supplementary Table 1. Human amniotic epithelial cells ameliorate kidneydamage in ischemia-reperfusion mouse model of acute kidney injury. [file 13287_2020_1917_MOESM4_ESM.docx]

**Human amniotic epithelial cells ameliorate kidney damage in ischemia-reperfusion mouse model of acute kidney injury**

Yifei Ren^123#^, Ying Chen^123#^, Xizi Zheng^1234^, Hui Wang^1235^, Xin Kang^123^, Jiawei Tang^123^, Lei Qu^1234^, Xiaoyan Shao^7^, Suxia Wang^1235^, Shuangling Li^6^, Gang Liu^1234^, Li Yang^1234*^

Supplementary Table 1. Primers for mouse growth factors and macrophage markers.

| **Gene** | **Primer sequence** |
| --- | --- |
| mEGF  mFGF  mVEGF  mHGF  mPDGF  mIGF-1  mF4/80  mIFN-γ  miNOS  mTNF-α  mCD86  mCD163  mCD206  mIL-4Rα  mArg-1 | F: 5′- GAGTTGCCCTGACTCTACCG-3′  R: 5′- CAATATGCATGCACACGCCA-3′  F: 5′- GGCTTAGCTCGAGACATTCATC-3′  R: 5′- CTCCCACAAGAGCACTCCAA-3′  F: 5′- TGTCAACGGTGACGATGATGG-3′  R: 5′- CTTGCAGATGTGACAAGCCAA-3′  F: 5′- CAATCCTGATGGCAAGCCGA-3′  R: 5′- ACATCAGTCTCATTCACAGCACT-3′  F: 5′- GCGTGTGACATTCCTGAACA-3′  R: 5′- ACACTGTCTCTTTGTCCTCGG-3′  F: 5′- GAAAATGCCACATCACCGCA-3′  R: 5′- GGGGGAAATGCCCATCTTTG-3′  F: 5′- TGGGATGCATAATCGCTGCT-3′  R: 5′- CCTCAGAACCCACAGTGTCC-3′  F: 5′- TGTTTGTGTGAAGGCCCTGT-3′  R: 5′- CCCAGGACCCAACACTTTGT-3′  F: 5′- CCCTTCAATGGTTGGTACATGG-3′  R: 5′- ACATTGATCTCCGTGACAGCC-3′  F: 5′- ATCCGCGACGTGGAACTG-3′  R: 5′- ACCGCCTGGAGTTCTGGAA-3′  F: 5′- TCCAAGAGCCACTCCTACCT-3′  R: 5′- AGTGCTGCCTGCTAAGTTGT-3′  F: 5′- GTGTGGGGTTGCCCAATCTA-3′  R: 5′- CCAGAGCAGTCATGAGGCAA-3′  F: 5′- TCTTTGCCTTTCCCAGTCTCC-3′  R: 5′- TGACACCCAGCGGAATTTC-3′  F: 5′- ATTCCCTGCCCTGCTGTTAC-3′  R: 5′- GATGGGACTGGTGAGGGTTG-3′  F: 5′- ACCTGGCCTTTGTTGATGTC-3′  R: 5′- CAGCACCACACTGACTCTTC-3′ |
